# Modelling of growth reaction pathways of zincone ALD/MLD hybrid thin films: a DFT study of precursor screening and the diethyl zinc pulse†

**DOI:** 10.1039/d5ra00686d

**Published:** 2025-05-15

**Authors:** Mario Mäkinen, Kari Laasonen

**Affiliations:** a Department of Chemistry and Materials Science, School of Chemical Engineering, Aalto University Kemistintie 1 02150 Espoo Finland kari.laasonen@aalto.fi

## Abstract

ALD/MLD hybrid thin films can be fabricated by combining atomic and molecular layer deposition (ALD and MLD). Density functional theory (DFT) can be used to determine the reaction paths of the growth reactions of these hybrid thin films. In this study, DFT was utilized to investigate the reaction mechanisms between diethyl zinc and 4-aminophenol using a surface model to examine the reactions responsible for the structure of the zincone thin film. The most feasible reaction path for the film growth was discovered. The effects of reconstructions, steric repulsion and functional group reactivity on the growth rate were also discussed in this study. Additionally, we compared the thin film growth reactions between diethyl zinc and thirteen organic bifunctional precursors using gas-phase calculations. This allowed us to create a trend for the reactivity of the precursors based on their functional groups, which can be used to aid in experimental precursor selection. We also established a connection between the bond strengths of the reacting precursors and the durability of the hybrid thin film in ambient conditions.

## Abstract 2

Growth reaction pathways were discovered for multiple zincone ALD/MLD hybrid thin films using DFT to investigate their composition and durability.

## Graphical abstract

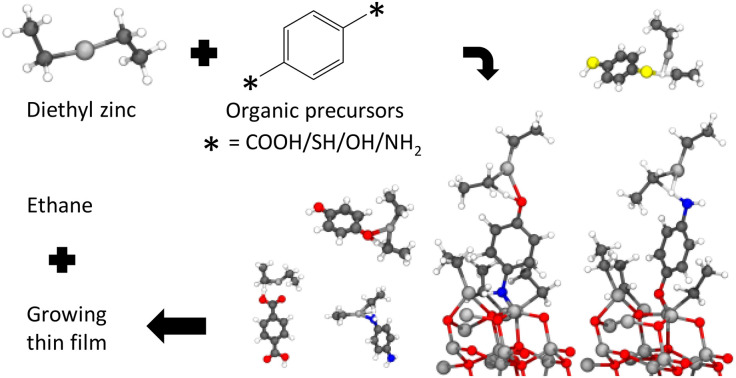

## 1 Introduction

Atomic Layer Deposition (ALD) is a widely-used chemical vapor deposition technique to fabricate ultrathin films with a thickness in the range of nanometers.^1,2^ ALD is essential in the semiconductor industry due to the ever-shrinking nature of electrical components.^1^ The main benefit of the method is the extremely smooth and conformal thin film structure, which can be applied on very large and uneven substrates, and on multiple substrates simultaneously in a parallel manner.^1,2^ However, the major drawback of ALD is its limited pool of precursors, which can be used to fabricate the product only from a limited set of inorganic materials. The product is usually some binary metal compound: an oxide, a sulfide or a nitride.^3^

Molecular layer deposition (MLD) is a method to fabricate polymer thin films, such as polyimides and polyamides.^3,4^ To add new functionalities for the thin film fabrication, ALD has been combined with MLD to create new possibilities for the precursor selection. By combining the methods, the hybrid thin films can be fabricated from a wide range of precursors, as they combine inorganic and organic layers in the structure of the thin film. For example, a thin film consisting of ZnO, a highly transparent and electrically conductive wide-bandgap semiconductor,^5^ can be fabricated using ALD with diethyl zinc and water as precursors, with a nitrogen pulse in between to discard the unreacted precursor molecules from the reaction chamber.^6^ By using ALD/MLD, organic components can be incorporated into the thin film structure using, for example, hydroquinone as a precursor.^6^ This way the properties such as elasticity, optical transmittance, and both thermal and electrical conductivity can be tailored to fit the application of the thin film.^5,6^ However, hydroquinone as a homobifunctional precursor suffers from growth hindering double reactions, *i.e.* reactions of both functional groups within a single precursor pulse.^5,7^ Thus, 4-aminophenol is recognized as an alternative to hydroquinone because as a heterobifunctional precursor the reactivity of the reacting functional groups should be different towards the other precursor, which should prevent double reactions from occurring. Additionally, the rigid backbone of aromatic 4-aminophenol should make it an ideal precursor to prevent double reactions from occurring.^7,8^

ALD/MLD can still be considered a black box method, as the reaction pathways that constitute the growth and the final structure of the thin film are still unknown. Computational methods, of which notably density functional theory, can be used to discover these reaction pathways. The benefits of DFT-methods are in process optimization, where we can research the structure and growth of the thin film, and finding factors that contribute to experimentally relevant phenomena. Additionally, computational methods can be used for screening of the precursors to find the most suitable precursor candidate for the given applications based on its reactivity with other precursors, and its properties, such as durability towards the ambient humidity. After the screening process, the most promising precursor candidates can be further researched through experimental work.

In this study, zincone (*e.g.* zinc-containing) hybrid thin films were examined using both gas-phase and surface models. The more computationally expensive surface reactions were used to study the diethyl zinc pulse of hybrid thin films fabricated using diethyl zinc and 4-aminophenol as precursors. We have studied the 4-aminophenol pulse earlier.^9^ The relatively computationally inexpensive gas-phase screening was used to investigate the reactivity of diethyl zinc with a variety of aromatic and aliphatic organic precursors.

### 1.1 Growth reactions of the ALD/MLD hybrid thin films

**Fig. 1:**
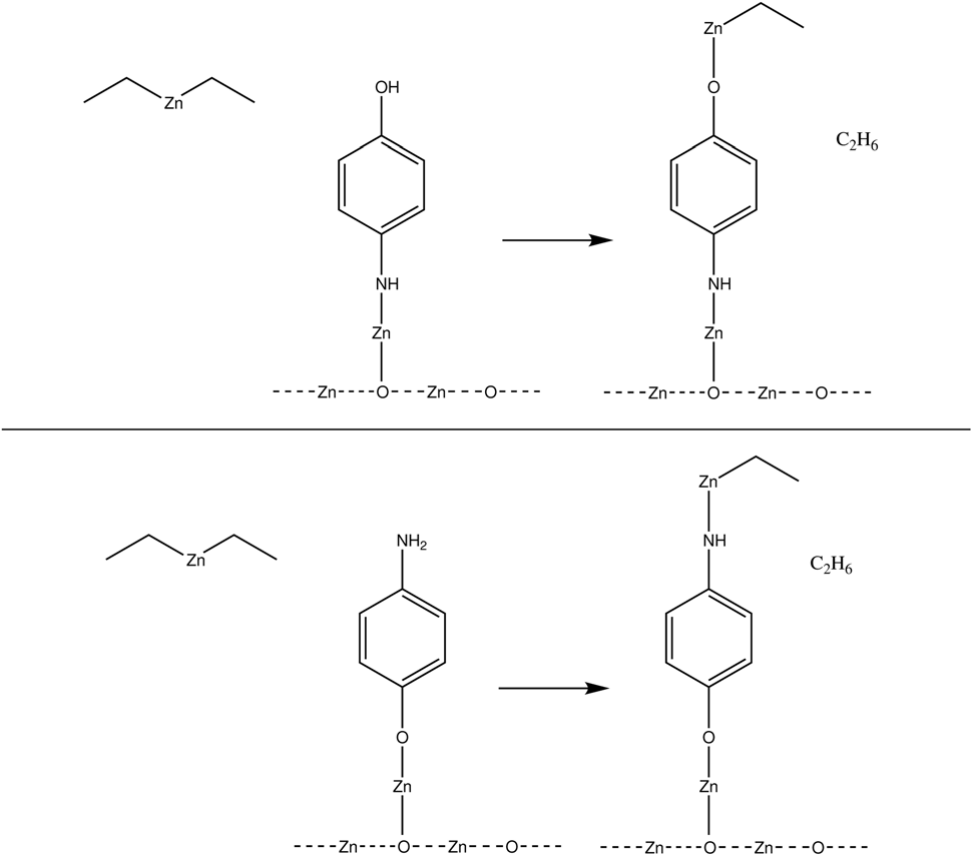
The common growth reactions during the diethyl zinc precursor pulse should follow a ligand-exchange reaction pathway. As the 4-aminophenol of the former organic precursor pulse can react using either of its functional groups, both reactions with the hydroxyl group (upper) and the amino group (lower) were required to be studied.

This study investigates the adsorption reactions occurring during the ALD/MLD growth process, where diethyl zinc and 4-aminophenol are used as precursors.^7^ The deposition process starts with ALD-cycles of zinc oxide deposition where water and diethyl zinc were used as precursors, followed by deposition of the first organic precursor pulse of 4-aminophenol. The first organic precursor pulse was studied earlier by our research group^9^ and this study focuses on the next precursor pulse, where diethyl zinc adsorbs on the surface. This hybrid thin film growth reaction should follow a ligand-exchange reaction pathway, for which two different configurations are presented in Fig. 1. The previously adsorbed 4-aminophenol can adsorb using either its hydroxyl or amino group, and therefore reactions with both functional groups were required to be studied.

The hybrid thin film growth reactions presented in Fig. 1 were studied using DFT. The reaction energy Δ*E* of these reactions was calculated as a subtraction of the energy of the initial state *E*_init_ from the energy of the reacted state *E*_fin_, which corresponds to the final state of the reaction. The activation energy *E*_act_ for the reaction between the precursors was calculated using the energy of the highest energy state, *i.e.* the transition state *E*_ts_ along the minimum energy path from the initial state to the final state of the reaction, and subtracting *E*_init_ from *E*_ts_.

The energy of the molecular adsorption Δ*E*_m.ads._ of the precursor molecule on the ethyl-saturated zinc oxide surface was calculated with eqn (1),

1 Δ*E*_m.ads._ = *E*_init_ − *E*_meox_ − *E*_adsorbate_

where *E*_init_ is the total energy of the molecular adsorption system which is also the initial state of the chemisorption reaction. *E*_meox_ is the energy of the 4-aminophenol- and ethyl-saturated zinc oxide slab, and *E*_adsorbate_ is the total energy of an independent molecule, which was diethyl zinc.

**Fig. 2:**
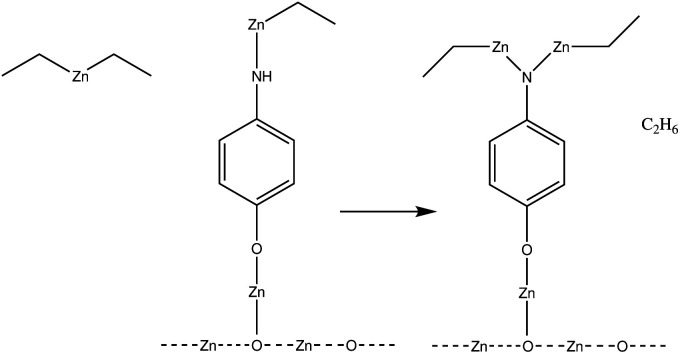
The reaction with an amino group should be more common within the second precursor pulse.^9^ As the amino group has two hydrogens, it can react twice with two different diethyl zinc precursors.

Based on our earlier results,^9^ the 4-aminophenol should adsorb on the surface hydroxyl group first. This allows two diethyl zinc precursor molecules to react with a singular 4-aminophenol in each deposition cycle without prohibitively high steric repulsion, as the amino group is the most easily available functional group and it has two hydrogen atoms which can both be donated to separate diethyl zinc precursors. The adsorption of this second diethyl zinc is presented in Fig. 2.

The adsorption energy Δ*E*_ads_ for the second diethyl zinc was calculated using eqn (2),

2 Δ*E*_ads_ = *E*_surf,dez_ − *E*_rmet_ − *E*_adsorbate_

where *E*_surf,dez_ is the energy of the system where ethane is removed from, and dissociated diethyl zinc is added to the final state of the ligand-exchange reaction presented in Fig. 1. *E*_rmet_ is the total energy of the system, where ethane is removed from the final state of that same ligand-exchange reaction. *E*_adsorbate_ is the total energy of an independent molecule, which was diethyl zinc.

**Fig. 3:**
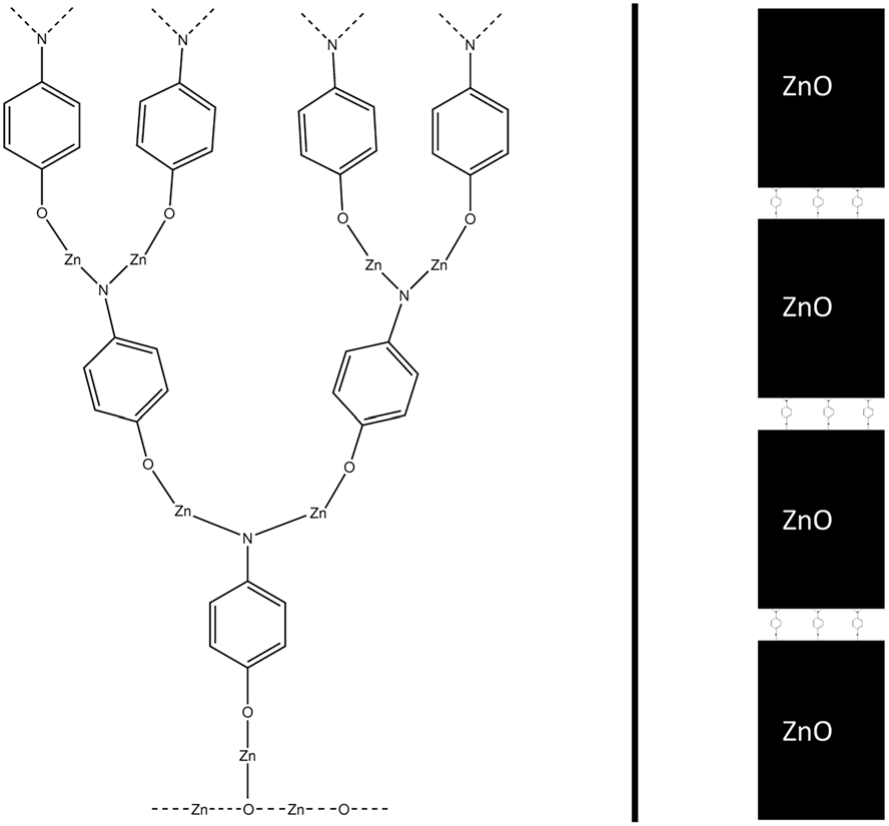
If each amino group reacts twice within a single diethyl zinc precursor pulse, this results in the thin film structure presented on the left, where the branching of the film increases over deposition pulses. This effect is hindered in superlattice structures,^10^ where a single organic layer is divided with thicker layers of zinc oxide.

The number of times a single amino group reacts can have an impact on the macrostructure of the hybrid thin film. If an amino group can react twice with separate diethyl zinc molecules, this can have a very noticeable impact on the structure of the hybrid thin film, as it enables horizontal growth, in addition to the vertical growth occurring due to alternating precursor pulses. This can lead to an ever-thickening thin film structure as the number of available sites for the adsorption of the next precursor pulse increases over deposition cycles. This effect should not be that noticeable in superlattice structures,^10^ where a single organic layer is separated with tens or hundreds of layers of zinc oxide, as the number of amino groups in the thin film structure is relatively limited. Both a conventional and a superlattice structure of a hybrid thin film are presented in Fig. 3.

**Fig. 4:**
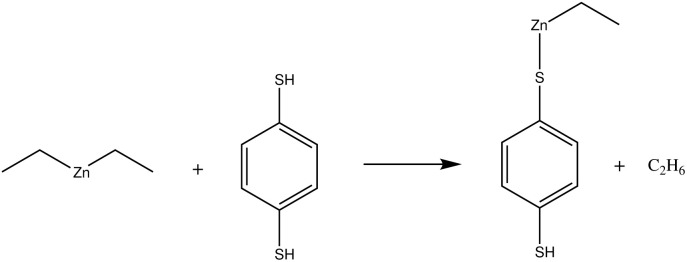
Ligand-exchange reaction between diethyl zinc and 1,4-benzenedithiol (BDT). As the gas-phase reactions are much less computationally demanding, ligand-exchange reactions can be calculated for multiple precursors, and thus their reactivity and bond strength can be compared with each other.

**Fig. 5:**
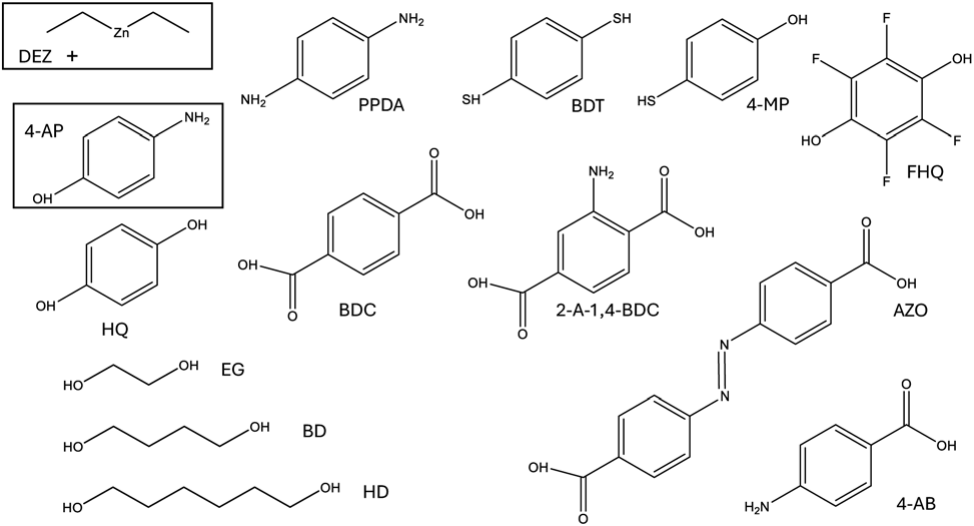
Along with the reactions between diethyl zinc and 4-aminophenol (in black rectangles) studied in the surface calculations, the ligand-exchange reactions between diethyl zinc and other organic molecules were studied in the gas-phase calculations.

In our earlier publication,^9^ we discovered that the ligand-exchange reaction is the most common reaction pathway in both gas-phase and surface calculations, and that activation and reaction energies are quite similar in both environments. As gas-phase reactions are computationally much less demanding, we can compare the reactivity of different organic precursors with diethyl zinc in a ligand-exchange reaction. As an example, the ligand-exchange reaction of 1,4-benzenedithiol with diethyl zinc is presented in Fig. 4. Fig. 5 includes all the precursors studied in this work and the full names and abbreviations of these precursors are presented in Table 1.

**Table 1:** Full names and abbreviations of all precursors used in this study

Abbreviation	Full name
DEZ	Diethyl zinc
4-AP	4-Aminophenol
PPDA	*p*-Phenylenediamine
BDT	1,4-Benzenedithiol
4-MP	4-Mercaptophenol
FHQ	Tetrafluorohydroquinone
HQ	Hydroquinone
BDC	Benzenedicarboxylic acid
2-A-1,4-BDC	2-Amino-1,4-benzenedicarboxylic acid
AZO	4,4′-Azobenzenedicarboxylic acid
EG	Ethylene glycol
BD	1,4-Butanediol
HD	1,6-Hexanediol
4-AB	4-Aminobenzoic acid

### 1.2 Experimental ALD/MLD hybrid thin film research

This study focuses on zincone hybrid thin films, and the surface reactions are studied for a thin film fabricated using diethyl zinc and 4-aminophenol as precursors. Such a thin film was fabricated by Sood *et al.*^7^ 4-Aminophenol is a heterobifunctional precursor with a rigid backbone. These properties should prevent the occurrence of unwanted double reactions, and thus make the thickness of the hybrid thin film directly proportional to the amount of precursor cycles. Sood *et al.* used deposition temperatures of 140–330 °C to fabricate these amorphous hybrid thin films. The growth rate stayed rather constant: 1.1 Å in the deposition temperature range of 140–200 °C. These hybrid thin films were reported to be stable only in relatively dry conditions, and that their durability towards humidity can be improved with few ALD-cycles of zinc oxide as the top layers of the thin film.

Zincone hybrid thin films can also be fabricated as a superlattice structure, where a single organic layer divides multiple layers of zinc oxide. Zincone superlattice containing 4-aminophenol has been fabricated by Tynell *et al.*^10^ They also fabricated zincone superlattices using either HQ or 4,4′-oxydianiline as a precursor in the same study. The ratio between organic and inorganic layers was between 199 : 1 and 49 : 1 for the fabricated thin films. The reaction temperature was 220 °C. The reaction rate for ZnO fabricated with ALD was 1.65 Å per cycle, and the change when organic layers were included in the structure was small. In room temperature measurements, the increasing amount of organic layers led to a non-linear decrease in the Seebeck coefficient and a non-linear increase in resistivity. Ghiyasi *et al.*^11^ fabricated zincone superlattice thin films, using PPDA, BDC, and 4,4′-oxydianiline and DEZ as precursors. Their objective was to fabricate a light material suitable for flexible thermoelectric applications. The deposition temperature was 220 °C. They found that the high thermal conductivity of zinc oxide can be lowered efficiently from 48 W m^−1^ K^−1^ to 12.7–3.3 W m^−1^ K^−1^ by including organic layers into the thin films structure.

The reactions responsible for the growth of the hybrid thin films should be sequential, and therefore double reactions are not preferential, as they can stop the growth of the thin film. Liu *et al.*^8^ investigated zincone hybrid thin films with either HQ or EG used as the organic precursor. The chemical composition of the hybrid thin film had a noticeable effect on the thermal conductivity of the thin film. For EG, the average growth rate was at most 0.86 Å, and for HQ the average growth rate was 2.73 Å. Due to double reactions, the average growth rate decreases for the thin films using EG as a precursor as the amount of cycles increases, but stays rather constant in the case of HQ. Also the density of reactive sites can be 98.1% for HQ-containing films, and 36.4% for EG**-**containing thin films. Due to the large number of double reactions, the deposition of EG-containing hybrid thin films that have a thickness of over 100 nm was reported to be rather challenging. The most common reason for the smaller amount of double reactions for the HQ-containing films in comparison to EG-containing films was reported to be the aromatic backbone of HQ, which will increase the energy required to rotate the precursor in comparison to EG. By utilizing crystal microbalance measurements with the thickness of the hybrid thin films, the significant amount of double reactions when using DEZ and EG as precursors was also observed by Peng *et al.*^12^

The choice of precursors can have a significant impact on the durability of the zincone hybrid thin films in ambient conditions. Peng *et al.*^12^ studied the hybrid thin films fabricated using DEZ and EG as precursors. The IR-spectrum revealed that the hybrid thin film is stable in dry air. However, by using IR, XPS and ellipsometry measurements, they discovered that ambient air will impair the structure of the thin film. Choudhury *et al.*^13^ studied the deposition of hybrid thin films in ambient conditions using HQ and DEZ as precursors in 150 °C deposition temperature. They discovered that the fabricated hybrid thin films can be made stable in ambient air by capping the thin film with a 20 nm layer of zinc oxide. Aghaee *et al.*^14^ studied the stability of the hybrid thin films fabricated using DEZ and HQ as precursors. They discovered, that the reason for the degradation of the thin film under humidity, according to ellipsometric porosimetry, is micro-porosity that enables the migration of the water of the humid air into the thin film structure. Five layers of zinc oxide were reported to be enough to prevent this migration. Philip *et al.*^15^ used bis-3-(*N*,*N*-dimethylamino)propyl zinc(ii) with HQ to fabricate hybrid thin films. In a relatively low deposition temperature of 60 °C, the growth rate was 3.2 Å per cycle. They demonstrated that a duration of two weeks in ambient conditions degraded the chemical composition of the thin film. This is a relatively long time when using a hydroxyl-containing precursor, as zincone hybrid thin films fabricated using DEZ and EG remain stable for one hour^12^ and DEZ and HQ for a day^14^ in ambient conditions. Philip *et al.*^16^ fabricated zincone hybrid thin films using non-pyrophoric bis-3-(*N*,*N*-dimethylamino)propyl zinc instead of DEZ, with BDT as another precursor, so that the thin film deposition can be conducted at a lower deposition temperature, and the pyrophoricity of DEZ can be avoided. This type of zincone thin film holds a promise to be an excellent barrier layer for temperature sensitive and flexible electronic devices. The thin films were electrically isolating, and a growth rate of 4.5 Å per cycle was achieved at 60 °C deposition temperature. Fabricated thin films were appreciably stable in ambient air over extended storage periods. Khayyami and Karppinen^17^ fabricated photoresponsive thin films using DEZ, water and AZO as precursors. Photoisomerization of the hybrid thin film utilizes UV-light, and the kinetics of the *trans*–*cis* photoisomerization was somewhat dependent on the chemical structure of the thin film. These hybrid thin films were stable in ambient conditions.

### 1.3 Computational ALD/MLD hybrid thin film research

Our earlier research,^9^ focused on the reactions of diethyl zinc with 4-AP and HQ using both gas-phase and surface models. We discovered that the 4-aminophenol will react first with its hydroxyl group. When the oxide of the zinc oxide is in the immediate vicinity of the adsorbing 4-AP, as is the case in superlattice structures, 4-AP can donate its hydrogen to oxygen, which will slow down the release of ethane from the surface. We also discovered that the ligand-exchange reactions are quite similar in both gas-phase and surface reactions, when oxygen is not available for the reaction.

The computational research of zincone hybrid thin films has also been conducted by other groups. Kim *et al.*^18^ studied reactions between diethyl zinc and various homobifunctional aliphatic precursors using gas-phase models. The functional groups of these precursors, which were OH, SH, and NH_2_, were located at both ends of the carbon chain of a varying length. The aim of the study was to study reaction paths that lead to formation of zinc oxide. Kim *et al.* suggest that homobifunctional precursors with a long carbon chain are suitable for the fabrication of zinc oxide, and those with a short carbon chain are suitable to fabricate ALD/MLD hybrid thin films. They also suggest, that long carbon chain containing diamino compounds are valid precursors to fabricate zinc nitride thin films. Karttunen *et al.*^19^ investigated the bulk structure of crystalline HQ-containing zincone superlattice thin films. They built an atomic-level model of the bulk structure, and used it to examine the experimental IR-spectrum. They also demonstrate that the band structure of the thin film can be tailored by changing the chemical composition of the hybrid thin film. Choudhury *et al.*^20^ used gas-phase models to study the bond lengths of HQ-containing zincone hybrid thin films, and utilized the results in the interpretation of an IR-spectrum. They also discovered that adding zinc oxide between the organic layers will make the thin film structure more thermodynamically stable.

Other common ALD/MLD hybrid thin films studied computationally by other groups are titanicone (*i.e.* titanium containing) and alucone (*i.e.* aluminium containing) hybrid thin films. Tanskanen *et al.*^21^ investigated adsorption reactions of HQ, 4-AP and PPDA on a TiCl_3_-terminated TiO_2_ anatase (101)-surface. They discovered that bonds formed by hydroxyl groups are noticeably more stable than the bonds formed by amino groups. Muriqi and Nolan^22^ investigated reactions of TiCl_4_ and Ti(DMA)_4_ precursors with EG and glycerol using surface models with either Al_2_O_3_ or an anatase or rutile TiO_2_ as the substrate. They discovered that double reactions are thermodynamically feasible when EG and glycerol react with Ti(DMA)_4_. This can explain why the thin film deposition using EG and Ti(DMA)_4_ will halt prematurely, while the deposition with glycerol will not, as the glycerol has an additional hydroxyl group that can continue the deposition reaction. Muriqi *et al.*^23^ studied the methyl-terminated Al_2_O_3_-surface after the trimethyl aluminium pulse, which reacts with HQ, PPDA and 4-AP. They discovered that double reactions with these combinations of precursors seem unlikely as HQ, PPDA and 4-AP prefer an upright configuration, which makes the second reaction within the same precursor pulse unfeasible. Yang *et al.*^24^ used gas-phase models to research the reactions of trimethyl aluminum with 4-AP, PPDA, 4-dinitrobenzene, 4-nitroaniline, and 4-nitrophenol. They discovered that reactions of trimethyl aluminum are exothermic, but the removal of a methyl group from trimethyl aluminum requires energy to overcome the reaction barrier. They also discovered that reactions of trimethyl aluminum are significantly slower when the reacting functional group of the other precursor is NH_2_ or NO_2_ than when the functional group is OH.

## 2 Computational methods

This study focuses on the modelling of growth reaction pathways of zincone ALD/MLD thin films. Modelling of these reaction pathways was conducted using GPAW^25^ with Atomic Simulation Environment.^26^ Density functional theory (DFT) was used with the Perdew–Burke–Ernzerhof (PBE) exchange and correlation functional.^27^ Van der Waals correction TS09, by Tkatchenko and Scheffler,^28^ was utilized to account for the weak interactions between a large aromatic precursor and ethyl ligands, which can have a noticeable contribution to the reaction energy of the system. Dipole corrections were not applied in our calculations. The criterion for convergence was the force smaller than 0.05 eV Å^−1^ on all individual atoms, which offers a good balance between accuracy and computational efficiency. For the entropy calculations, the converge criteria for the force was tightened to 0.01 eV Å^−1^ and the convergence criteria for the maximum change in eigenstates was set to 10^−8^ eV^2^ per valence electron. All of the reaction barriers were calculated using the nudged elastic band method with a climbing image,^29^ utilizing a chain of 10 images of the system and a FIRE^30^ optimizer. The climbing image algorithm was used to find the saddle point *i.e.* the transition state from the minimum energy path acquired with the nudged elastic band method. Even though the use of a hybrid functional could be beneficial, especially for the accuracy of the activation energy calculations,^31^ they are computationally too expensive to be utilized in this study. The Visual Molecular Dynamics^32^ program was used in the visualization of the results.

In our surface calculations, real-space grid spacing was 0.2 Å, and the sampling of the *k*-space was conducted with a 3 × 3 × 1 Monkhorst–Pack grid. The lowest layer of the zinc oxide slab was constrained in its place. Earlier we discovered^9^ that even though the band gap description of zinc oxide could benefit from using the Hubbard+*U* correction,^33^ it has only a minute effect on the reaction energies and was therefore excluded from our calculations. The steric repulsion caused by the ethyl ligands that originate from the diethyl zinc precursor has a significant effect on the total energy of the system. To minimize the effect of this repulsion, a combination of molecular dynamics (MD) and geometry optimization was used in the search for the structure that would minimize this repulsion, resulting in a lower total energy for the studied surface system. 3 picosecond MD simulations with 1 femtosecond time steps were used to probe the potential energy surface. Then, geometry optimization was used for generated structures between 500 femtosecond intervals to find an energy minimum for the given surface structure. Due to the large number of rotational degrees of freedom of the ethyl ligands present in the studied ethyl-saturated zinc oxide surfaces, this process was repeated starting from the newly discovered low energy structure, until the optimization of MD-generated structures did not yield a structure with lower total energy. The lowest energy structure was then used as a starting point for the CI-NEB calculations. A small polarized double-*ζ* basis set was used in MD simulations. Temperature was kept at a constant 550 K using a Berendsen thermostat. This temperature is still within the deposition range of the studied Zn–AP hybrid thin film,^7^ so that unwanted decomposition reactions can be prevented in the short time frame of MD, while the relatively rapid movement of ethyl ligands should expedite the discovery of a lower total energy structure used in the CI-NEB calculations.

## 3 Results

### 3.1 Surface reactions

As a benchmark for our computational model, we used experimental work by Sood *et al.*^7^ Their hybrid thin film consisted of alternating cycles of diethyl zinc and 4-aminophenol, with an initial base layer of 40 ALD cycles consisting of diethyl zinc and water. To model the initial ALD base layer, we used computational work by Weckman and Laasonen,^34,35^ which considers the incomplete growth of the thin film,^36^ which is dependent on the deposition temperature of the thin film. This temperature dependence is modelled as two different surface slab structures denoted case 1 and case 2, which correspond to deposition temperatures of approximately 100–140 and 140–180 °C, respectively. This temperature range provides a good representation of the thin film growth, as the experimentally verified ALD window of 140–200 °C is followed by decrease in growth rate at higher temperatures, and finally a drastic decrease in film quality at 280 °C.^7^

#### 3.1.1 Case 1 structure

The case 1 surface by Weckman and Laasonen^34,35^ was constructed in the following way. Four water molecules were dissociated on three layers of (ZnO)_8_, forming a hydroxylated (100) zinc oxide surface. This surface reacted with five diethyl zincs using a ligand-exchange reaction presented in eqn (3). Five diethyl zincs were chosen because a higher concentration than five monoethyl zincs made the structure unstable. Five ligand-exchange reactions remove five hydrogen atoms from the surface, making the chemical formula for the case 1 surface (ZnO)_24_(ZnC_2_H_5_)_5_O_4_H_3_.^34^

3 ‖–Zn–OH + Zn(C_2_H_5_)_2_ ⇒ ‖–Zn–O–Zn(C_2_H_5_) + C_2_H_6_

In our earlier work,^9^ we studied the adsorption of the first organic precursor pulse on the surface, in the form of an adsorbing 4-aminophenol molecule. The adsorbing 4-aminophenol can adsorb using its amino or hydroxyl group and utilizing either a ligand exchange or a dissociation reaction. However, despite the reacting functional group or the reaction pathway, the end result is always the reaction presented in eqn (4), where 4-aminophenol (OHC_6_H_4_NH_2_) reacts with the surface using either of its functional groups. The structures used to model the adsorption of 4-aminophenol can be acquired from the Harvard Dataverse.^9,37^

4 ‖–Zn–C_2_H_5_ + OHC_6_H_4_NH_2_ ⇒ ‖–Zn–(OC_6_H_4_NH_2_) + C_2_H_6_

**Fig. 6:**
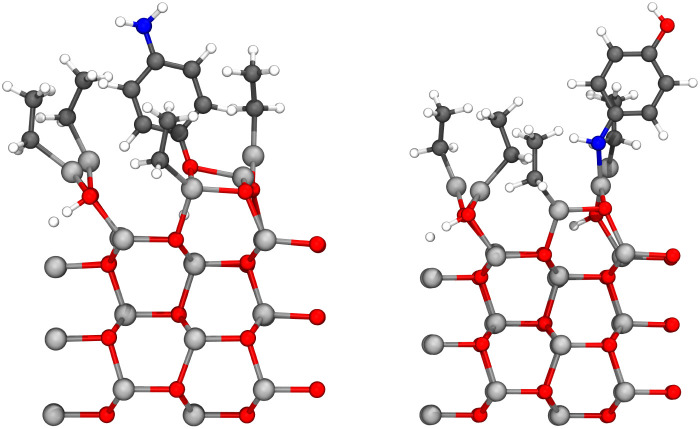
The study of the diethyl zinc pulse was started from the slab structure, where 4-aminophenol has adsorbed to the surface from the organic pulse utilizing either its hydroxyl group (left) or amino group (right), and the by-product ethane is removed from the surface. These structures correspond to a thin film deposition conducted at the lower deposition temperature (denoted case 1), which corresponds to a higher ethyl-ligand concentration than in case 2. The lone hydrogen is bonded, but the bond cannot be seen due to the periodicity of the unit cell. The colors of atoms in the figure: nitrogen in blue, oxygen in red, hydrogen in white, zinc in light grey, and carbon in black.

After the reaction, the purging nitrogen pulse removes ethane (C_2_H_6_) from the reaction chamber, which was accounted for by removing the ethane from the surface structure. This leads to a structure with a chemical formula (ZnO)_24_(ZnC_2_H_5_)_4_(C_6_H_6_NO)ZnO_4_H_3_. Finally, the structures need to be stabilized to minimize steric repulsion on the surface, which was done using the method described in Section 2 Computational methods. This will lead to two different structures where the 4-aminophenol is adsorbed utilizing either its amino or hydroxyl group. These structures are presented in Fig. 6. The size of both unit cells was 6.73 × 10.48 × 36.77 Å.

**Fig. 7:**
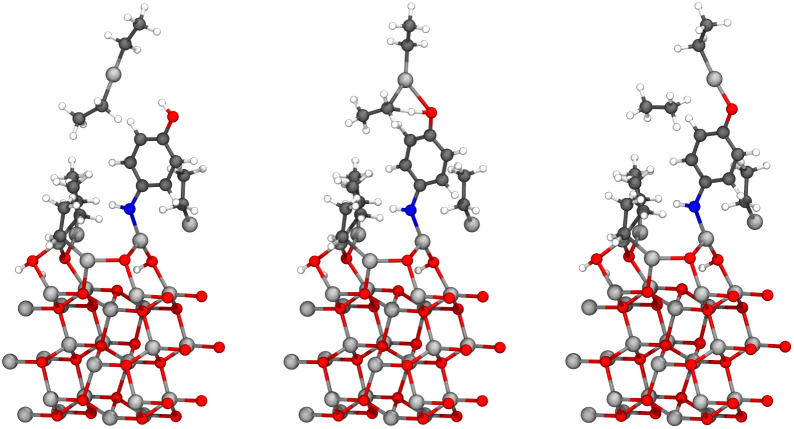
The reaction between diethyl zinc and the hydroxyl group of the adsorbed 4-aminophenol proceeded as expected leading to a growing hybrid thin film. Initial state is presented on the left, transition state in the middle, and the final state on the right. The lone monoethyl zinc is bonded, but the bond cannot be seen due to the periodicity of the unit cell. The colors of atoms are the same as in Fig. 6. The transition state is stabilized by partial bonds between the donated hydrogen, zinc, carbon and oxygen, of which only some are shown in the figure.

**Fig. 8:**
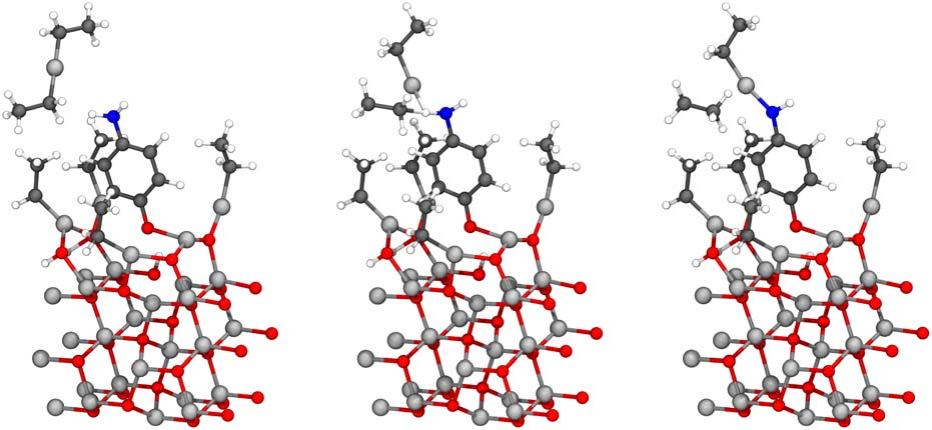
The reaction between diethyl zinc and the amino group of the adsorbed 4-aminophenol proceeded as expected leading to a growing hybrid thin film. Initial state is presented on the left, transition state in the middle, and the final state on the right. The colors of atoms are the same as in Fig. 6. The transition state is stabilized by partial bonds between the donated hydrogen, zinc, carbon and oxygen, of which only some are shown in the figure.

By adding diethyl zinc and increasing the height of the unit cell to 40 Å due to the vacuum region otherwise being only 5.84 Å high above the hydrogen of the hydroxyl group and 7.29 Å high above the hydrogen of the amino group, both structures in Fig. 6 can be used to study the adsorption of the diethyl zinc during the second precursor pulse. The lowest layer of the zinc oxide slab was constrained in its place throughout the calculations. Based on our previous results,^9^ 4-aminophenol favors energetically adsorption with the hydroxyl pointing down, hence the reaction between the amino group and diethyl zinc should be significantly more common during this precursor pulse. As the surface is saturated with diethyl zinc and the steric repulsion on the surface is relatively high, the only feasible pathway should be a ligand exchange reaction between diethyl zinc and the yet-unreacted functional group of the adsorbed 4-aminophenol. This reaction is presented in Fig. 7 for the hydroxyl group, and in Fig. 8 for the amino group.

The ligand exchange reaction proceeded as expected: monoethyl zinc and hydrogen are the exchanging ligands of the reaction, thus resulting in a thin film growing end product and an ethane molecule, which is expected to be removed in the next purging phase. Activation energies *E*_act_, reaction energies Δ*E* and molecular adsorption Δ*E*_m.ads._ of these reactions are presented in Table 2.

**Table 2:** Calculated energies for reactions between diethyl zinc and the adsorbed 4-aminophenol on the case 1 surface. The values located in the first three rows are the reaction parameters of the first diethyl zinc, and the adsorption energy located in the last row is for the second diethyl zinc

Calculated energies (eV)	Amino	Hydroxyl
Δ*E*_m.ads._	−0.25	−0.48
Δ*E*	−0.57	−0.79
*E*_act_	1.27	1.06
Δ*E*_ads_	−1.02	

**Fig. 9:**
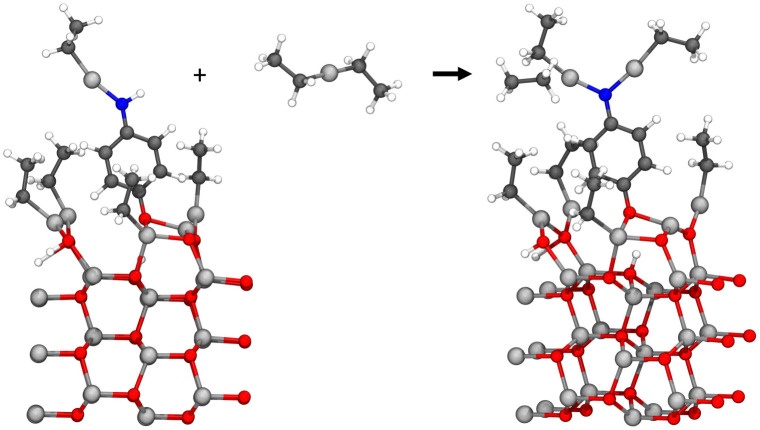
As the amino group has two hydrogens, a second diethyl zinc can also react with 4-aminophenol within the same precursor pulse. The adsorption energy for this reaction was calculated using eqn (2).

The oxygen-first adsorbed 4-aminophenol has two hydrogens available in its amino group, which enables also a second diethyl zinc to react with it within a single precursor pulse. This reaction is presented in Fig. 9. It is presumed that the ethane from the earlier reaction is not present in the surface, even though the purging of ethane has not yet occurred. The adsorption energy Δ*E*_ads_ of −1.02 eV makes this reaction spontaneous when compared to the loss of entropy for the reaction, which we have estimated to be 0.2 eV using the difference in entropy between freely moving and gaseous diethyl zinc, and freely moving and gaseous ethane along with adsorbed monoethyl zinc in the harmonic limit at 140 °C temperature. The adsorption energy is included in Table 2.

#### 3.1.2 Case 2 structure

The case 2 structure by Weckman and Laasonen^34,35^ was hydroxylated similarly to the case 1 structure, with 4 dissociating water molecules on three layers of (ZnO)_8_, forming a hydroxylated (100) zinc oxide surface. However, at higher deposition temperature an additional ligand exchange reaction is available on the surface, which creates bare zinc atoms, and is presented in eqn (5). It was assumed that all the available hydrogen on the surface was consumed in ligand exchange reactions presented in eqn (3) and (5). The most stable structure contained a total of two bare zinc atoms and four monoethyl zinc groups, and therefore the chemical formula of the case 2 surface is (ZnO)_24_(ZnC_2_H_5_)_4_Zn_2_O_4_.^34^

5 ‖–Zn–O–Zn(C_2_H_5_) + ‖–Zn–OH ⇒ (‖–Zn–O)_2_–Zn + C_2_H_6_

**Fig. 10:**
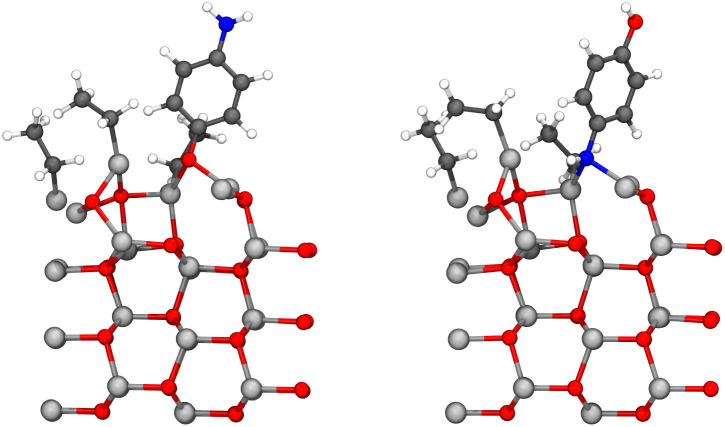
The study of the diethyl zinc pulse was started from the slab structure, where 4-aminophenol has adsorbed to the surface from the organic pulse utilizing either its hydroxyl group (left) or amino group (right), and the by-product ethane is removed from the surface. These structures correspond to a thin film deposition conducted at the higher deposition temperature (denoted case 2), which corresponds to a lower ethyl-ligand concentration than in case 1. The lone monoethyl zinc is bonded, but the bond cannot be seen due to the periodicity of the unit cell. The colors of atoms are the same as in Fig. 6.

As in the case 1 structure, the adsorbing 4-aminophenol can adsorb using its amino or hydroxyl group and utilizing either a ligand exchange or dissociation reaction.^9^ Similarly, the reaction will follow eqn (4), followed by the removal of ethane due to the nitrogen pulse used in the experiments to remove ethane from the reaction chamber. The chemical formula for the structures after the ethane removal is (ZnO)_24_(ZnC_2_H_5_)_3_(C_6_H_6_NO)Zn_3_O_4_. The steric repulsion was minimized utilizing the method described in Section 2 Computational methods, and the results are presented as two structures in Fig. 10. Similar to the case 1 structures, the size of both unit cells was 6.73 × 10.48 × 36.77 Å.

**Fig. 11:**
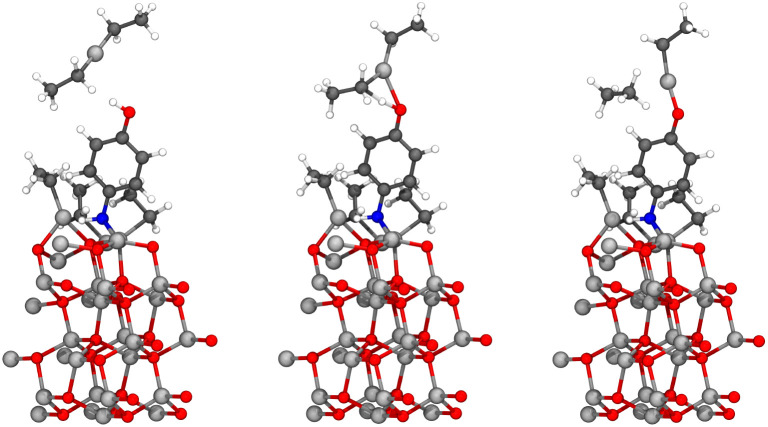
The reaction between diethyl zinc and the hydroxyl group of the adsorbed 4-aminophenol proceeded as expected leading to a growing hybrid thin film, and elimination of the ethyl ligand as gaseous ethane. This reaction is relatively rare, as 4-aminophenol energetically favors reacting with the hydroxyl group first. Initial state is presented on the left, transition state in the middle, and the final state on the right. The colors of atoms are the same as in Fig. 6.

**Fig. 12:**
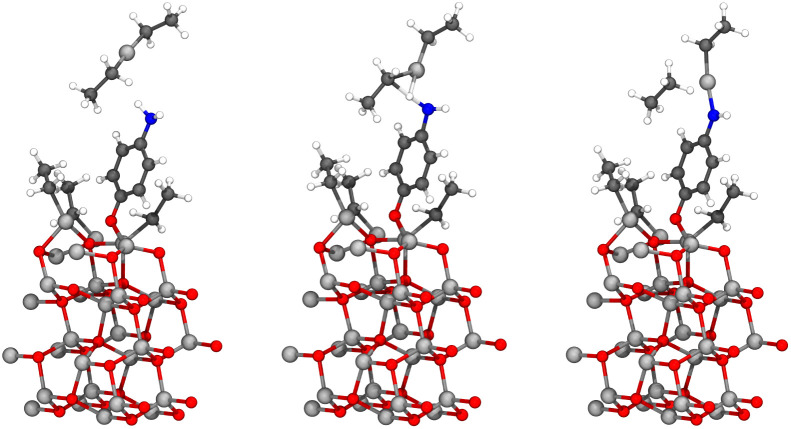
The reaction between diethyl zinc and the amino group of the adsorbed 4-aminophenol proceeded as expected leading to a growing hybrid thin film, and elimination of the ethyl ligand as gaseous ethane. This reaction is common, as 4-aminophenol energetically favors reacting with the hydroxyl group first. Initial state is presented on the left, transition state in the middle, and the final state on the right. The colors of atoms are the same as in Fig. 6.

The structures presented in Fig. 10 were used to study the diethyl zinc pulse by including the diethyl zinc molecule into the unit cell and increasing the height of the unit cell to 40 Å, due to the vacuum region otherwise being only 6.85 Å high above the hydrogen of the hydroxyl group and 6.58 Å high above the hydrogen of the amino group. The lowest layer of the zinc oxide slab was constrained in its place throughout the calculations. Based on our earlier results,^9^ 4-aminophenol reacts also on the case 2 surface first with its hydroxyl group, and therefore the more common reaction is the reaction between the amino group of 4-aminophenol and diethyl zinc. These ligand exchange reactions proceeded as expected: diethyl zinc will donate its monoethyl group to the adsorbed 4-aminophenol to grow the hybrid thin film, whilst 4-aminophenol will donate its hydrogen to form ethane, which will be removed in the next purge. The reactions are presented in Fig. 11 for the hydroxyl group and in Fig. 12 for the amino group. Activation energies *E*_act_, reaction energies Δ*E* and molecular adsorption Δ*E*_m.ads._ of these reactions are presented in Table 3.

**Table 3:** Calculated energies for reactions between diethyl zinc and the adsorbed 4-aminophenol on the case 2 surface. The values located in the first three rows are the reaction parameters of the first diethyl zinc, and the adsorption energy located in the last row is for the second diethyl zinc

Calculated energies (eV)	Amino	Hydroxyl
Δ*E*_m.ads._	−0.18	−0.21
Δ*E*	−0.63	−0.89
*E*_act_	1.13	0.83
Δ*E*_ads_	−1.17	

**Fig. 13:**
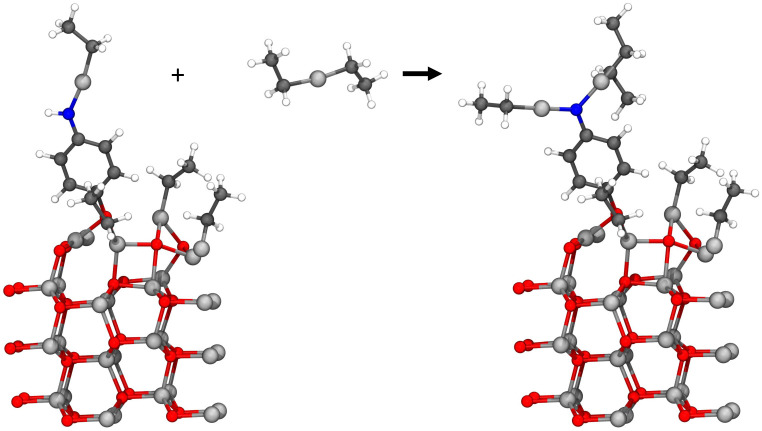
As the amino group has two hydrogens, a second diethyl zinc can also react with 4-aminophenol within the same precursor pulse. The adsorption energy for this reaction was calculated using eqn (2).

Similar to the case 1 structure, the amino group of the adsorbed 4-aminophenol can react with two diethyl zinc molecules, when it has adsorbed to the surface using its hydroxyl group. Conversely, the second reaction with the amino group would be prevented by strong steric repulsion, if the 4-aminophenol had reacted amino group first. The reaction of the second diethyl zinc is presented in Fig. 13. This reaction is spontaneous, as the adsorption energy of −1.17 eV is stronger than the 0.2 eV entropy opposing the reaction. The adsorption energy is included in Table 3.

### 3.2 Gas-phase screening

**Fig. 14:**
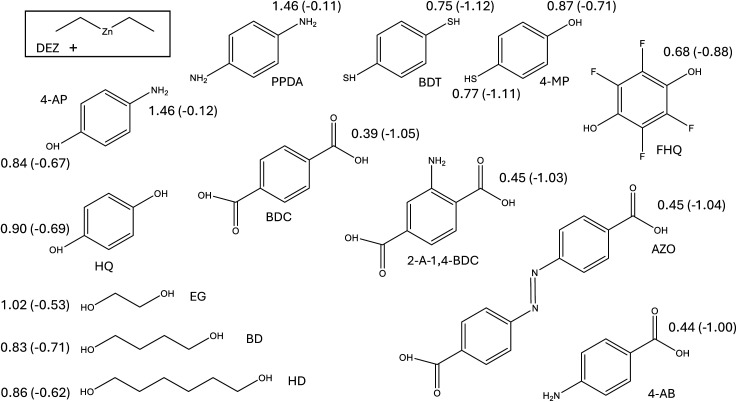
The calculated activation and reaction energies for the different functional groups of different organic precursors in their reactions with diethyl zinc. The reaction energies are presented in parenthesis after the activation energies. The unit of energy values is eV. The calculated energies for HQ and 4-AP are adopted from our previous work.^9^

**Fig. 15:**
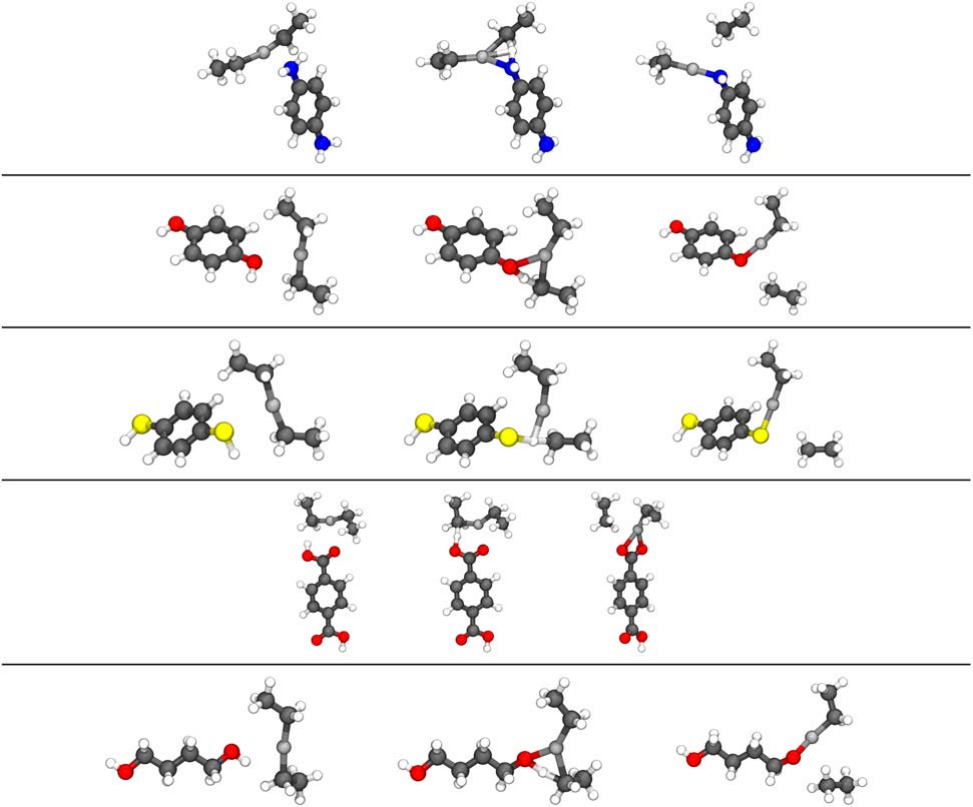
Initial state (left), transition state (middle), and final state (right) of the ligand exchange reaction between DEZ and PPDA, HQ, BDT, BDC or BD (in this order from top to bottom) in the gas phase. The colors of atoms in the figure: nitrogen in blue, hydrogen in white, zinc in light grey, carbon in black, oxygen in red and sulfur in yellow.

Modelling of the ligand exchange reactions responsible for the growth of different zincone hybrid thin films was conducted using computationally relatively inexpensive gas-phase calculations. Studied gas-phase reactions consisted of diethyl zinc reacting with one of the organic precursors presented in Fig. 14, where they are presented along with the calculated activation and reaction energies. If the reacting functional groups of the precursors were different, both were investigated. The only difference was 4-AB, where the large difference between energies of the functional groups, amino and carboxyl groups, led to converge issues when studying the reaction with significantly higher activation energy, which was the one with the amino group. Reacting functional groups of the studied organic precursors were amino, hydroxyl, thiol, and carboxyl, of which example reactions are presented in Fig. 15 as reactions of PPDA, HQ, BDT and BDC, with BD as an example of an aliphatic alcohol.

## 4 Discussion

### 4.1 Surface model

#### 4.1.1 The growth of the hybrid thin film

Based on the surface calculations, diethyl zinc would prefer to react with the hydroxyl group rather than the amino group of 4-aminophenol on both the first^9^ and the second precursor pulse. This is why diethyl zinc will most often react with the amino group of the 4-aminophenol during the diethyl zinc pulse, as most of the 4-aminophenol used its hydroxyl group to react with the monoethyl zinc during the 4-aminophenol pulse.^9^ Due to the absence of nearby oxygen of the zinc oxide layers during the diethyl zinc pulse, the reaction between the amino group and diethyl zinc follows the only feasible pathway, which was a ligand exchange pathway. Even though rare in the diethyl zinc pulse,^9^ also the reaction with the hydroxyl group follows a ligand exchange reaction pathway.

Due to the amino group having two N–H bonds, there is a possibility for two reactions of a single amino group with two different diethyl zinc precursors within the same precursor pulse. This is supported by the lack of N–H bonds present in an FTIR-spectrum of the final structure of the film reported by Sood *et al.*, along with the presence of an aromatic ring with C–N and C–O bonds, and the absence of O–H bonds. In addition, the FTIR-spectrum by Sundberg *et al.*^38^ confirmed the presence of Zn–N and Zn–O bonds. This combination of requirements for the structure is fulfilled by amino groups reacting twice. According to our results, the second reaction of the amino group with diethyl zinc is both exothermic and spontaneous, as the adsorption energy (−1.02 and −1.17 eV in the case 1 and 2 surfaces, respectively) is stronger than the entropy of 0.2 eV at 140 °C. Additionally, the adsorption energies of −1.02 and −1.17 are slightly higher than the addition of the energies of molecular adsorption and the reaction energy of the first reaction between the amino group and the diethyl zinc, which were −0.82 and −0.81 eV on the case 1 and case 2 surface, respectively. Therefore it is quite feasible, that the amino group will react with both of its hydrogens. The most viable reaction pathway should be a ligand exchange reaction with the diethyl zinc.

As the hydroxyl group is the more reactive functional group of the 4-aminophenol, both of the monoethyl zincs, which were attached to the amino group in a previous diethyl zinc pulse, should react with the hydroxyl group within the next 4-aminophenol pulse, and the amino group of this 4-aminophenol should in turn react with two diethyl zincs in the next diethyl zinc pulse. Therefore a conventional hybrid thin film should thicken as the deposition proceeds, as due to the amino groups, the amount of available adsorption sites and thus reacting diethyl zincs should increase during every deposition cycle. There is a possibility that steric repulsion can hinder this effect. However, it should be noted that if fan-like growth is hindered by repulsion, it would lead to unreacting N–H bonds, which are absent from the IR-spectrum.^7^ The fan-like growth should not affect drastically the structure of superlattice structures, as the number of organic precursor pulses is drastically smaller than in conventional thin films.

After 4-AP from the very first organic precursor pulse has adsorbed on the surface, the growth of the conventional hybrid thin film should be rather straightforward, as it should consist solely of ligand exchange reactions. Due to the absence of available oxygen originating from an ALD water pulse, there is not a possibility for the dissociation reactions, which were present in the first precursor pulse,^9^ and led to a trapping of the reaction within its intermediate reaction steps, thus lowering the reaction rate. However, the energies of molecular adsorption (−0.18 to −0.48 eV) are noticeably smaller than the entropy of gaseous diethyl zinc (1.6 eV at 140 °C), thus leading to a small amount of diethyl zinc approaching the surface. In addition, as the activation energy of the reaction between the amino group of 4-aminophenol and diethyl zinc is relatively high (1.27 and 1.13 eV) and the amino group has to react with diethyl zinc to grow the thin film, the growth of the hybrid thin film should be relatively slow in the diethyl zinc pulse, as it was in the first 4-AP pulse. This is supported by the reported growth rate of 1.1 Å per cycle at 140–200 °C temperature,^7,38^ which is significantly lower than the 7 Å growth that we have approximated as the ideal average growth of the thin film in each deposition cycle based on the distance between atoms in the calculated structures. For reference, Liu *et al.*^8^ approximated the ideal growth rate to be 8.4 Å for a hybrid thin film fabricated using diethyl zinc and hydroquinone, which has a similar structure to 4-aminophenol apart from one functional group being a hydroxyl group instead of an amino group.

The slow growth rate is an exchange for the homogeneous growth of the film, as the heterobifunctionality of 4-AP should prevent unwanted double reactions occurring on the surface,^7,8,12^ when heterobifunctional 4-aminophenol is being used as a precursor. This effect occurs likely due to the low reactivity of the amino group, which will lead to adsorption sites being filled mostly with the hydroxyl groups instead of amino groups. Then, the low reactivity of the amino group can be compensated in the diethyl zinc pulse by, for example, using longer pulsing times, and thus preventing the occurrence of double reactions, while still enabling the growth of the thin film. This effect was demonstrated for 4-aminophenol and diethyl zinc in this study, but it should be duplicated with other heterobifunctional precursors with a noticeable difference in the reactivities of the functional groups.

A possible cause for the less than one monolayer growth rate would be reconstructions occurring during the thin film deposition process. The fan-like growth presented in this study supports these reconstructions, as the constantly thickening structure would cause a lot of stress on the lower bonds of the thin film structure. According to our calculations, we suggest that the final structure of the hybrid thin film is noticeably influenced by steric repulsion, fan-like growth and reconstructions.

#### 4.1.2 Proposal for a full-cycle reaction mechanism

**Fig. 16:**
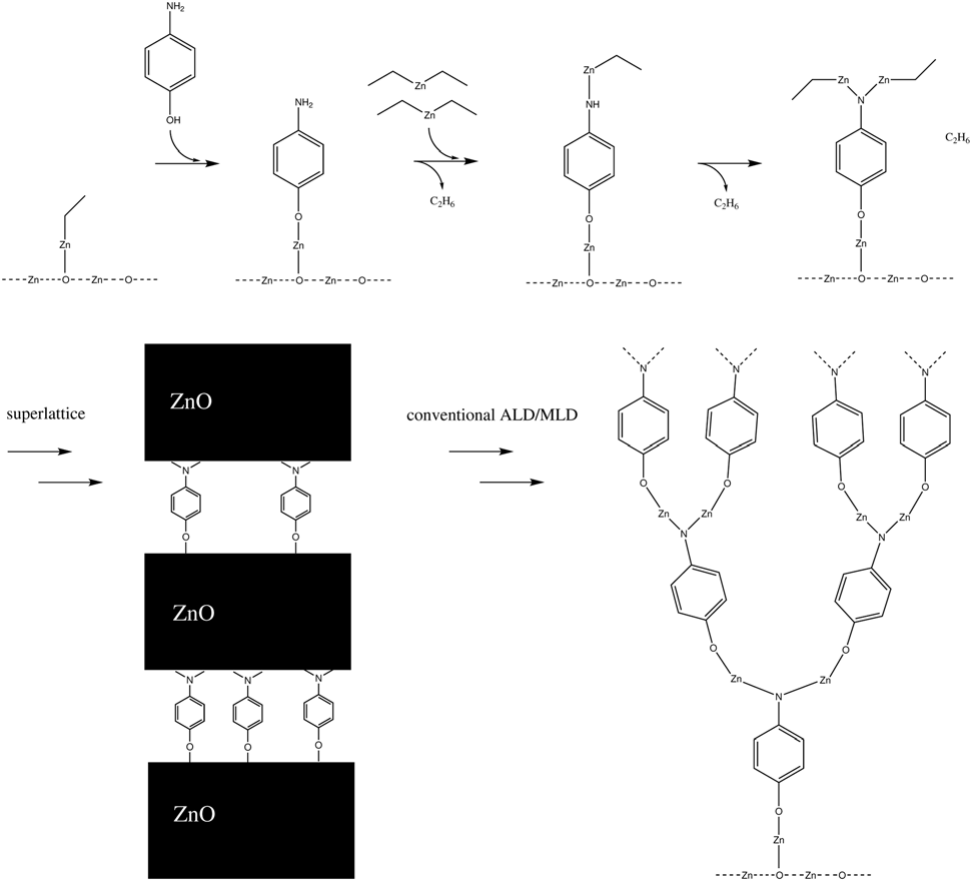
A schematic of the proposed full-cycle reaction mechanism of ALD/MLD growth using DEZ and 4-AP as precursors (on top). In a superlattice structure (bottom left), this cycle is followed by multiple ZnO-layers from ALD-cycles of water (next pulse) and DEZ. In a conventional ALD/MLD hybrid thin film, the ALD/MLD cycle is repeated, but the first reaction with 4-AP occurs with twice the amount of monoethyl zinc, due to the amino group reacting twice during the diethyl zinc pulse. This will lead to a branching structure (bottom right), which should undergo periodical reconstructions due to the stress this type of growth would cause to the chemical bonds.

By combining the data from this and our earlier study,^9^ we can propose the most feasible reaction path for the hybrid thin film growth. This reaction path is presented in Fig. 16. If the structure of the hybrid thin film is a superlattice, such as in the experimental work by Sundberg *et al.*,^38^ the reaction path continues with a water pulse followed by a diethyl zinc pulse. This ALD-cycle is repeated, until an organic layer is added to the surface periodically using the reaction path presented in Fig. 16. Both the water and diethyl zinc pulses of an ALD-cycle have been studied computationally by Weckman and Laasonen.^34,35^

For a conventional hybrid thin film, such as in the experimental work by Sood *et al.*,^7^ the suggested reaction path is repeated starting with a ligand exchange reaction between monoethyl zinc and 4-aminophenol. Due to the amino group reacting twice during the diethyl zinc pulse, the amount of reactive sites is doubled after each cycle, which should cause branching growth of the film. However, this reaction cycle should be unsustainable by itself, due to the stress branching growth would cause to the chemical bonds of the thin film structure. In addition, branching growth can cause steric repulsion for the structure. Therefore, we propose periodic reconstructions occurring during the film growth.

#### 4.1.3 Further research

Due to the repulsion playing such a key role in the total energy of the system, it would be beneficial if the surface slab could be larger in size, and therefore the concentration of 4-aminophenol in the surface could be defined more accurately. This would enable the study of the adsorption of multiple precursor molecules within the same unit cell. A larger slab could also aid in the discovery of possible reconstructions occurring during the hybrid thin film growth, which could also increase the number of researchable precursor cycles on the surface. However, a larger surface slab would also mean an increase in the total number of monoethyl ligands on the surface, which makes the search of a low energy minimum using the approach of combining molecular dynamics with structure optimization at periodic intervals, unfeasible. One approach would be to use machine learning methods^39–41^ to expedite this search of minima, and thus possibly enable the use of a larger surface slab.

### 4.2 Gas-phase screening

The study of the growth reactions responsible for the growth of hybrid thin films was significantly cheaper computationally when it was conducted as a gas-phase calculation instead of surface calculation, thus enabling the screening of multiple precursors. However, these two methods are complementary to each other, as overall the structure of the thin film is amorphous.^7^ Therefore, the rigid crystal lattice structure of surface calculations describes better the earlier precursor pulses near the layers of zinc oxide of either the conventional or superlattice structure, whilst amorphous polymer-like growth present in later precursor pulses can be described better using a gas-phase model.

The studied gas-phase reactions were ligand exchange reactions which, based on the surface calculations with diethyl zinc and 4-aminophenol, are responsible for the majority of the growth reactions of the conventional hybrid thin film. Reactions proceeded as expected and the reaction paths were noticeably similar to each other. The majority of the studied precursors were bifunctional aromatic molecules, but also aliphatic bifunctional alcohols were under investigation. For aromatic precursors, the functional groups responsible for the growth of the hybrid thin film were located at the 1- and 4-positions of the aromatic ring. The functional groups responsible for the growth of the aliphatic alcohols were located at the ends of the carbon chain.

#### 4.2.1 Reactivity of the precursors

The calculated energies presented in Fig. 14 can be used to compare the relative reactivity of the studied precursors with DEZ. Whether the precursor was smaller in size like 4-AP or larger like AZO, the functional groups in the 1- and 4-positions of the studied aromatic precursors had only a small effect on the reactivity of each other. This makes aromatic compounds great benchmark molecules for the growth of different hybrid thin films. As the reactivity of both functional groups is so independent, we can acquire a trend of reactivities of different functional groups. The most reactive functional group is clearly carboxyl whilst the least reactive one is clearly an amino group. The thiol group is more reactive than hydroxyl according to precursors with homobifunctional functional groups (BDT and HQ), but this difference becomes less noticeable when comparing heterobifunctional compounds (4-AP and 4-MP). The 4-MP precursor shows an intramolecular difference between the reactivity of these functional groups. Additionally, thiol forms stronger bonds than the hydroxyl group according to the reaction energies. The better reactivity of the thiol group in comparison to the hydroxyl group is also supported by experimental results by Philip *et al.*,^42^ who used bis-3-(*N*,*N*-dimethylamino)propyl zinc as the zinc containing precursor, and propose that the significantly higher growth rate of BDT instead of HQ is due to the higher reactivity of BDT. When comparing FHQ with HQ, we can see that the fluorination of the benzene ring increases the reactivity and strengthens the bonding. When comparing 2-A-1,4-BDC with BDC, we discover that the amino group in the 2-position of the benzene ring weakens the reactivity of the carboxyl group in the 1-position slightly, but this difference becomes negligible when the comparison is extended to AZO and 4-AB.

Based on the reactivities of aliphatic alcohols presented in Fig. 14, there does not seem to be a considerable difference between the reactivities of hydroxyl groups, which are bonded to varying lengths of aliphatic carbon chains. There also doesn’t seem to be a significant difference between the reactivity of aliphatic and aromatic alcohols, which can also hold true regarding different functional groups. These trends are supported computationally by Kim *et al.*,^18^ who discovered that, for the reaction between adsorbed diethyl zinc as monoethyl zinc and aliphatic homobifunctional precursors with different lengths of carbon chains, the activation energies stay similar when varying the length of the carbon chain. They also found the reactivity of thiol, hydroxyl and amino groups to follow the same order that was found for the aromatic compounds in this study. Additionally, Yemini *et al.* discovered experimentally^43^ that when cysteine, which is an aliphatic compound including carboxyl, amino and hydroxyl groups, is reacting with DEZ it will not use its amino group in the reaction, but prefers the use of the thiol and carboxyl groups. This discovery is in agreement with the results presented in this study.

When comparing reaction energies of functional groups presented in Fig. 14 with experimental work, we discover that the relatively high reaction energy of −1.00 eV or higher (*i.e.* thiol and carboxyl group) is likely to form a stable hybrid thin film in ambient air,^17,42^ whereas a reaction energy of equal or less than −0.71 eV (hydroxyl and carboxyl group) seems unstable.^7,12–15^ This phenomenon seems feasible, as the likely cause of this instability is the unwanted reactions with the water of the ambient air,^12^ which can be prevented by the stronger bonds of the carboxyl and thiol groups. This provides a framework for the possible use of the reaction energy as an indicator for the durability of hybrid thin films in ambient conditions. As the reaction energy is computationally relatively inexpensive to calculate, this would be an efficient method to pre-screen precursors before experimental research in the case that the application requires a stable hybrid thin film in ambient conditions.

## 5 Conclusions

The growth reactions of zincone hybrid thin films were studied using computational methods based on density functional theory. The calculated energies of these growth reactions were utilized to investigate the structure of the hybrid thin films. A surface model was used to study the reaction between 4-AP and DEZ during the DEZ pulse of the hybrid thin film deposition process. 4-AP reacts first with its hydroxyl group during the 4-AP cycle,^9^ and therefore the reaction during the DEZ pulse is between DEZ and the amino group of 4-AP. The high activation energy of this ligand exchange reaction is likely a component of the slow growth rate of the thin film.^7^ An amino group of 4-AP has two N–H bonds which are both absent from the IR-spectrum^7^ of the hybrid thin film. Based on our results, we suggest that the two N–H bonds of 4-AP react with two different DEZ precursors within a single DEZ pulse. This reaction pathway leads to an ever-thickening thin film, as the number of available adsorption sites increases with each precursor cycle. However, this type of fan-like growth of the film is unfeasible by itself due to the high stress it would inflict on the lower layers of such a structure. To account for the unfeasible stress of the forming hybrid thin film structure, we suggest that reconstructions contribute to the final structure of the thin film, in addition to the fan-like growth and the steric repulsion likely caused by this type of growth.

Gas-phase ligand exchange reactions were used to study the reactions between DEZ and bifunctional organic precursors containing either carboxyl, thiol, hydroxyl or amino groups in their structure. Activation energies were then utilized to compare the reactivities of these functional groups. According to these activation energies, a carboxyl group reacts the fastest, an amino group the slowest, and a thiol group is slightly more reactive than a hydroxyl group. The 1- and 4-positions of an aromatic ring are relatively unaffected by the functional group in the other position, which makes these types of bifunctional aromatic compounds excellent benchmark molecules for the screening of ALD/MLD precursors. The carbon chain length of the aliphatic monobifunctional alcohols has no significant effect on the reactivity of the functional groups, and the reactivity of aliphatic alcohols was similar to the aromatic alcohols, which can also hold true for other functional groups. The reaction energies seem to correlate with the durability of the hybrid thin film in ambient conditions. This correlation makes screening a feasible method to gather information about the durability of a variety of hybrid thin films without the need for experimental laboratory work.

## Data availability

The data supporting this article have been included as part of the ESI† as xyz files. Data for this article, including both xyz and traj files are available at Harvard Dataverse at https://doi.org/10.7910/DVN/WFVEHM.^37^

## Author contributions

Mario Mäkinen: conceptualization, methodology, software, validation, formal analysis, investigation, data curation, writing – original draft, writing – review & editing, visualization, funding acquisition. Kari Laasonen: conceptualization, resources, writing – review & editing, supervision, validation, project administration, funding acquisition.

## Conflicts of interest

There are no conflicts to declare.

## Supplementary Material

RA-015-D5RA00686D-s001
